# Sophorolipid-Based Biosurfactant Application for Carbonated Water Flooding: An Experimental Approach for Enhanced Oil Recovery

**DOI:** 10.1021/acsomega.5c09824

**Published:** 2025-12-09

**Authors:** Praveen Singh, Gaurav Rawat, Darshan Halari, Amit Saxena, Shivanjali Sharma, Geetha S. J., Sanket J. Joshi, Prashant Jadhawar, Shital Khot

**Affiliations:** † Amity Institute of Microbial Technology, 211579 Amity University Rajasthan, Jaipur 303002, Rajasthan, India; ‡ Department of Petroleum Engineering and Geo-Engineering, 199276 Rajiv Gandhi Institute of Petroleum Technology, Jais 229304, India; § Amity Microbial Culture Center, Amity University Rajasthan, Jaipur 303002, Rajasthan, India; ∥ School of Engineering, 1019 University of Aberdeen, Aberdeen AB24 3UE, United Kingdom; ⊥ SNF FLOPAM India Pvt. LTD., Survey No. 141-1/2 & 142, NH 8A East, Kutch, Anjar 370240, Gujarat, India

## Abstract

Enhanced oil recovery (EOR) technologies are facing growing pressure to strike a balance between production efficiency and environmental sustainability. This study investigated sophorolipid (SL) biosurfactants produced by *Candida bombicola* as environmentally compatible alternatives to synthetic surfactants in carbonated water flooding applications. Comprehensive characterization using Fourier transform infrared (FTIR) and NMR spectroscopy confirmed a glycolipid structure with hydrophilic sophorose disaccharide heads and hydrophobic fatty acid tails. The biosurfactant demonstrated exceptional surface activity with a critical micelle concentration of 250 ppm, reducing the surface tension from 72 to 31 mN/m and achieving an ultralow interfacial tension of 2.2 mN/m, representing an 85% reduction from baseline conditions. The wettability modification experiments showed a systematic transition from oil-wet (106°) to water-wet (48°) conditions, facilitating enhanced oil mobilization. Synergistic effects were observed when 250 ppm of sophrolipid was combined with 1500 ppm of polyacrylamide (PAM), producing optimal shear-thinning rheology and a remarkable CO_2_ absorption capacity (2.82 mol/kg), 77% higher than that of water alone. High-pressure interfacial tension measurements confirmed the stability under CO_2_-enriched conditions (2.18 mN/m). These results establish SL-based biosurfactants as viable game changers for next-generation sustainable EOR operations, offering superior biodegradability, reduced toxicity, and the dual benefits of enhanced oil recovery with a CO_2_ sequestration potential.

## Graphical abstract

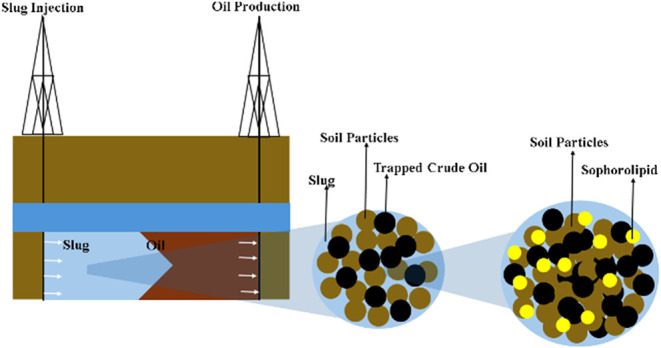

## 1 Introduction

Enhanced oil recovery (EOR) represents a pivotal technological advancement in petroleum engineering, designed to extract residual oil that remains after primary and secondary recovery operations have been completed. As conventional oil reserves become increasingly scarce and global energy demand continues to escalate, EOR techniques have gained critical importance in the petroleum industry.^1^ These advanced recovery methods can substantially enhance hydrocarbon extraction efficiency, increasing the recovery rates from the typical 30–40% achieved through conventional methods to as high as 60%.^2,3^

The urgent requirement for improved EOR technologies as two-dimensional nanomaterials has gained massive attention in the recent era of discoveries because of their unique properties, as well as is further intensified by the dual challenge of maximizing resource utilization while minimizing environmental impact.^4^ As the petroleum industry faces growing pressure to adopt sustainable practices, there is an increasing demand for environmentally compatible EOR solutions that maintain production efficiency without compromising ecological integrity. Contemporary EOR methods utilize advanced techniques to mobilize and extract trapped oil from subsurface formations. Among these, chemical flooding is one of the most widely employed techniques, utilizing surfactants and polymers to enhance fluid mobility and displacement efficiency.^5^ Surfactants, characterized by their amphiphilic molecular structure with both hydrophilic and hydrophobic components, reduce the interfacial tension between the oil and water phases through preferential adsorption at the liquid–liquid interfaces. Carbon dioxide flooding has emerged as a significant EOR method, in which CO_2_ injection reduces oil viscosity through miscibility effects while maintaining reservoir pressure.^6^ Furthermore, microbial enhanced oil recovery (MEOR)^7^ exploits indigenous or introduced microorganisms to produce biosurfactants in situ, facilitating improved oil mobilization.^8^

Although CO_2_ flooding demonstrates significant recovery potential, it presents substantial environmental challenges when injected CO_2_ is not permanently sequestered in the reservoir, leading to increased greenhouse gas emissions.^9^ This environmental concern has intensified the search for sustainable alternatives that can maintain production efficiency while reducing carbon footprint. Although the technical efficacy of chemical flooding with synthetic surfactants has been demonstrated in both laboratory and field settings, these methods pose significant environmental challenges that warrant attention. Conventional chemical surfactants, although effective in reducing interfacial tension and enhancing oil recovery, are characterized by poor biodegradability, high toxicity, and considerable potential for bioaccumulation in natural ecosystems.^3,10^ These synthetic compounds, commonly used in various industrial applications, persist in the environment after application, potentially causing long-term ecological harm to both aquatic and terrestrial ecosystems. The environmental persistence of synthetic surfactants has elicited serious concerns regarding their extensive use, particularly in subsurface environments where monitoring and remediation efforts are inherently difficult.^11,12^ Consequently, there is an urgent demand for environmentally benign alternatives that can achieve comparable performance without the associated ecological risks.

Biosurfactants offer a promising solution to the environmental challenges posed by conventional enhanced oil recovery (EOR) chemicals. These naturally occurring surface-active molecules, produced by various microorganisms, form a diverse group capable of reducing the surface and interfacial tension while playing crucial roles in microbial biology and metabolism.^3,13^ Their derivation from renewable biological resources makes them superior alternatives to synthetic surfactants from an environmental sustainability perspective. The advantages of biosurfactants include significantly reduced toxicity, enhanced biodegradability, improved ecological compatibility, and excellent stability under extreme conditions, such as high temperatures, varying pH levels, and elevated salinity.^14^ These characteristics render them particularly suitable for petroleum production environments, where harsh conditions involving high temperature, pressure, and chemical complexity are prevalent. Beyond their primary application in oil recovery, biosurfactants demonstrate versatility in various petroleum industry^15^ applications, including oil spill remediation, produced water treatment, tank cleaning operations, and specialized industrial processes such as emulsion polymerization for coatings and paints.^16,17^ Biosurfactants are classified into several major categories based on their chemical structures: glycolipids, lipopeptides, lipoproteins, phospholipids, fatty acids, polymeric surfactants, and particulate surfactants. Each class exhibits unique properties and characteristics that optimize its performance for specific applications and environmental conditions.^14,17^

Among the various biosurfactant classes, glycolipid biosurfactants have shown the most promising results for EOR applications. These molecules, which are produced by diverse microorganisms, exhibit exceptional performance characteristics in reservoir environments. SL has emerged as a particularly effective biosurfactant for enhanced oil recovery (EOR) applications.^18^ These glycolipid compounds are synthesized by nonpathogenic yeasts, primarily *Candida bombicola*, which was initially isolated from bumblebee honey. The production of SL by nonpathogenic organisms offers significant safety advantages over rhamnolipids, which are produced by potentially pathogenic bacteria such as *Pseudomonas aeruginosa*.^19^ SLs are recognized as highly promising natural surfactants in the petroleum industry owing to their consistent performance in EOR applications. These compounds are typically employed during tertiary recovery operations following the exhaustion of conventional primary and secondary methods. The unique molecular structure of SL, characterized by various structural analogues, isomers, and lactone configurations, contributes to its effectiveness as a surface-active agent.^20^

Several key properties render SL suitable for EOR applications.

- This excellent emulsification capacity facilitates the mixing of oil and water phases and enables the extraction of trapped oil from porous rock formations.^14,21^

- A Low critical micelle concentration (CMC) renders them economically viable for large-scale field applications.^22,23^

- The wettability alteration capability converts reservoir rocks to more water-wet conditions that favor enhanced oil displacement.^23,24^

- Emulsion stabilization ensures smooth oil transport through reservoir systems.^17,25^

- Exceptional stability under severe reservoir conditions; maintains surface-active properties despite the high temperature, pressure, and salinity.^16,20^

The economic and environmental advantages of SL make them appealing alternatives to traditional enhanced oil recovery (EOR) methods. Their cost-effectiveness, coupled with their environmental compatibility, positions them as viable solutions for sustainable oil recovery. The scalability of SL production can be augmented using renewable carbon sources, such as industrial waste streams and agricultural byproducts, thereby further enhancing their economic profile while supporting the principles of a circular economy.^23,26^ An innovative methodology involves integrating SLs with CO_2_ flooding techniques, resulting in a synergistic system that enhances oil recovery while facilitating carbon sequestration. This combined approach not only improves recovery efficiency but also contributes to CO_2_ storage, thereby preventing atmospheric release and supporting climate change mitigation efforts.^9^

This study examined the performance characteristics of SLs produced by *Candida bombicola* in enhanced oil recovery (EOR) applications. This research encompasses a comprehensive evaluation of critical parameters, including biosurfactant stability under varying operational conditions (pH, temperature, and salinity), surface and interfacial tension measurements, wettability alteration analysis of reservoir rocks, rheological behavior under reservoir-like conditions, adsorption characteristics on sand surfaces, and core flooding experiments to assess recovery efficiency. The primary objective was to demonstrate the technical feasibility and environmental advantages of the SL-based EOR while providing quantitative data to support field-scale implementation. This research addresses the critical need for sustainable EOR technologies that can reduce environmental impact while maintaining or improving oil recovery performance, thereby contributing to the development of more environmentally responsible petroleum production practices.

## 2 Experimental Section

### 2.1 Materials

SL-based biosurfactants were produced by microbial fermentation. Pusher 1000 (PAM) water-soluble polymer supplied by SNF FLOPAM India Pvt. Ltd. was used. All experiments were conducted using deionized (DI) water with an electrical conductivity of 0.0054 mS cm^–1^ to eliminate experimental interference from dissolved ions. A magnetic stirrer (Remi Lab-10MLH PLUS) was used to dissolve the polymer in DI water completely. For the CO_2_ absorption studies, highly pure carbon dioxide (99.99%) was obtained from Sigma Gases and Services, India. Crude oil used in this study was obtained from a leading oil transportation company in India. The crude oil properties were provided in a previous study.^6^

### 2.2 Methods

#### 2.2.1 Synthesis and Characterization of SLs

SLs, a class of glycolipid biosurfactants, are produced by certain nonpathogenic yeast strains, such as *C. bombicola*, under controlled environmental conditions. The process was carried out in two main stages:

##### 2.2.2.1 Seed Culture Preparation

The seed culture for biosurfactant synthesis is prepared by culturing the microbe in a nutrient-rich medium optimized for cell density and metabolic activity. The medium consisted of 2% glucose, 1% yeast extract, 1% peptone (GYP), and 2% waste frying oil. To prepare the seed media, 50 mL of GYP is autoclaved in 250 mL Erlenmeyer flasks and inoculated with *C. bombicola*. The flasks are then incubated for 48 h at 25 °C with agitation at 160 rpm. This fosters microbial adaptation, resulting in a high-density culture for larger fermentation batches. Using waste frying oil reduces costs and supports sustainability in biosurfactant production.^14,27–29^

##### 2.2.2.2 Preparation of Production Media

The production medium for biosurfactant synthesis is enriched with 3% glucose, 1% yeast extract, 1% peptone, and 5% waste frying oil. After the medium was sterilized, 5% of the seed culture was inoculated to ensure a high cell density. The flasks are incubated at 27 °C while being shaken at 160 rpm for 120 h. Samples are taken every 24 h to analyze optical density, pH, emulsification index (*E*_24_), and biosurfactant production using the oil spreading method.^3,8^

SL were extracted and characterized to evaluate their quality, and optical density measurements indicated strong microbial growth with peak values of 2.8–3.4 over a 120 h fermentation period. The pH values of the biosurfactant solutions ranged from 5.8 to 6.4. The emulsification efficiency, measured using the *E*_24_ index, reached 68–74% at concentrations above 500 ppm. The oil spreading test revealed effective surface tension reduction, with clear zones of 3.8–5.2 cm diameter at 1000 ppm.^30^ These findings confirmed that SL are high-quality biosurfactants suitable for possible applications in enhanced oil recovery, Figure 1.

**Figure 1:**
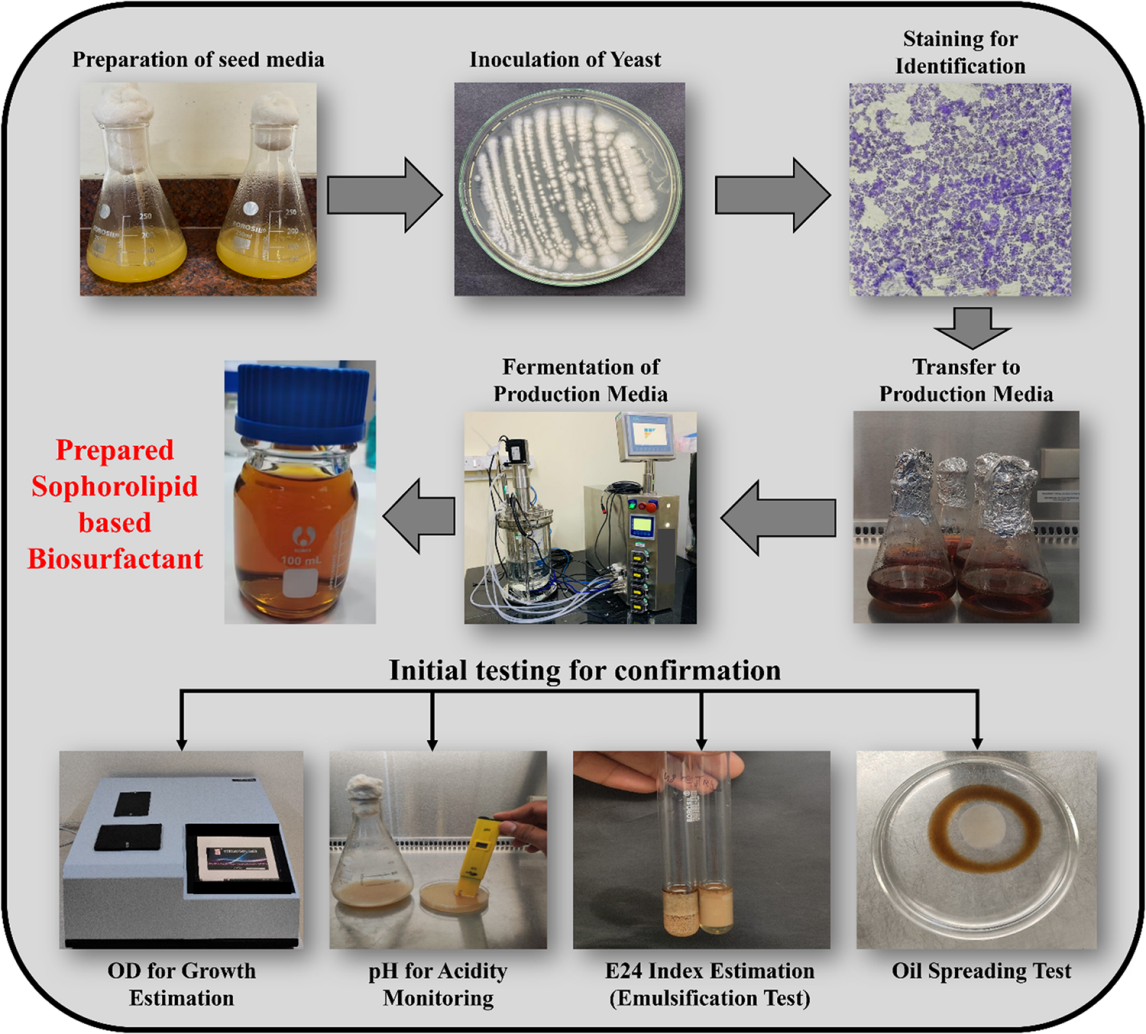
Production andd isolation of sophorolipids.

Fourier transform infrared (FTIR) spectroscopy is a rapid and valuable analytical technique used for determining the functional groups in a sample. The partially purified fractions were investigated using an FTIR spectrometer (PerkinElmer) in the wavenumber range of 400–4000 cm^–1^.^31^ For spectral analysis, high-purity analytical-grade potassium bromide (KBr) was prepared into pellets in which a small aliquot of the test sample was carefully blended to form uniform pellets suitable for transmission measurements. Simultaneously with FTIR analysis, nuclear magnetic resonance (NMR) spectroscopy was used to determine the molecular structure of the purified SL. NMR spectra were recorded on a Cary Eclipse Agilent Technologies spectrometer at 25 °C in deuterium oxide (D_2_O) as the solvent to minimize any potential interference in the spectrum and enhance the fidelity of the acquisitions.^32^ The FTIR and NMR analyses allowed for a careful characterization of the chemical composition and structural aspects of SL.

#### 2.2.2 Critical Micelle Concentration (CMC) and IFT Determination

The critical micelle concentration (CMC) of the synthesized SL biosurfactant was determined using a tensiometer (Kyowa Scientific) to evaluate its surface and interfacial-active properties and optimize its application in fields such as enhanced oil recovery (EOR), detergency, and emulsification. CMC, a key parameter indicating the concentration at which surfactant molecules aggregate to form micelles, was measured through surface tension (ST) assessments using the Wilhelmy plate method. The tensiometer was calibrated, and the glassware was cleaned to ensure accurate measurements. Additionally, the interfacial tension between the SL-based solution and crude oil was calculated using the Du Noüy ring method.^33^

#### 2.2.3 Wettability Characteristics

Wettability is the property of a fluid adhering to a crude-oil-saturated rock surface, where a contact angle experiment is commonly performed to measure the wettability of a flat surface (rock surface). The goniometer setup shown in Figure 2 was used to calculate the contact angle, which was purchased from Apex Instruments (India) (Acam-NSC series). In this experiment, a surfactant solution was formulated, and a drop of the solution was incorporated into the oil-wetted rock surface by using a Hamilton syringe. Subsequently, the alteration in wettability of the oil-dipped core surface was recorded to evaluate the impact of the surfactant treatment.

**Figure 2:**
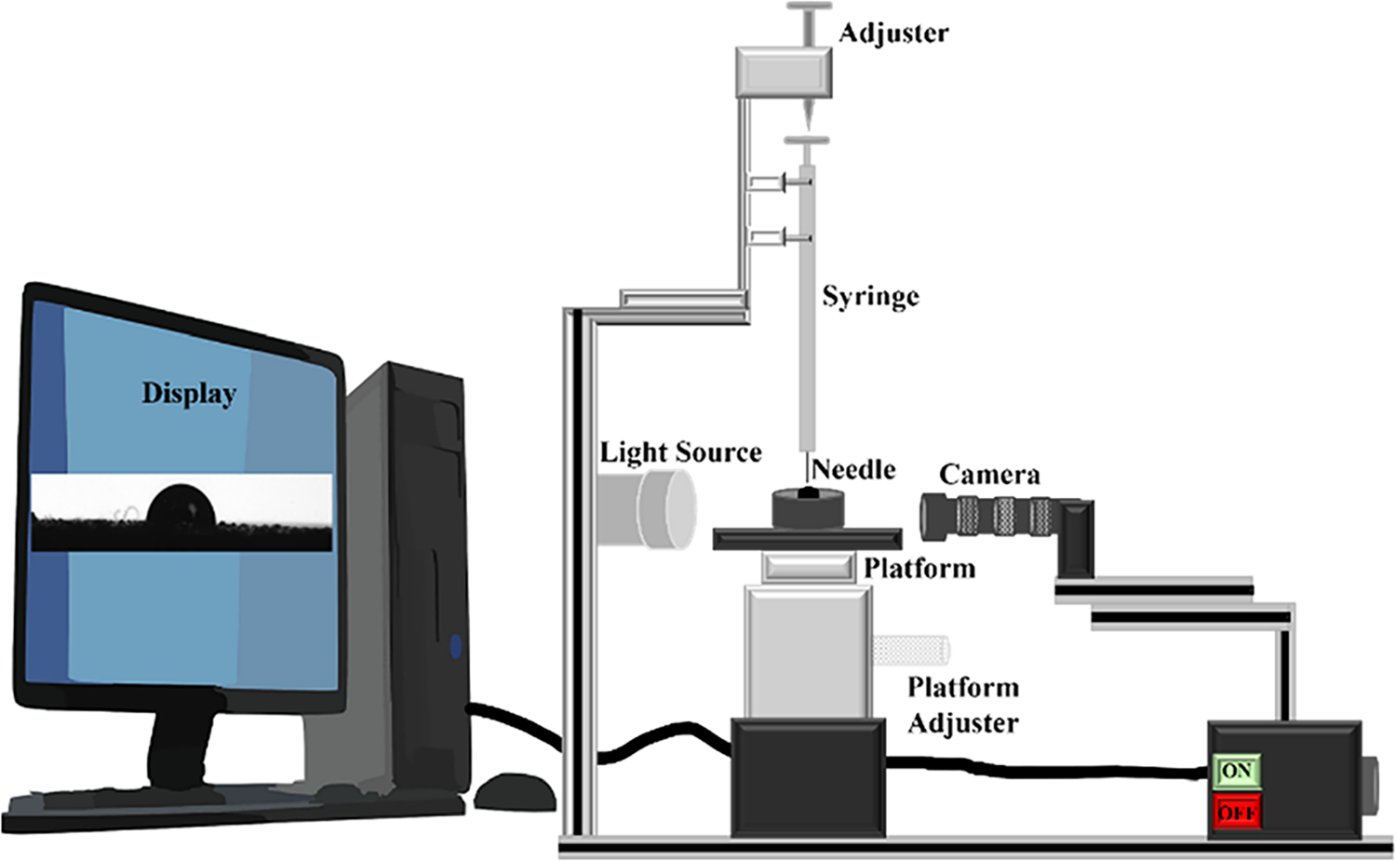
Schematic diagram for the contact angle experiment.

#### 2.2.4 CO_2_ Absorption Test

The CO_2_ absorption experiment was conducted in a high-pressure cell designed for the formulation of carbonated water. This cell, with a total volume capacity of 1300 mL, was engineered to withstand pressures of up to 4000 psi, ensuring safety and reliability during high-pressure operations. A measured volume of fluid was first introduced into the cell, which was then subjected to a vacuum for 10 min to eliminate any residual air or contaminants. Following evacuation, the empty cell was pressurized with high-purity carbon dioxide gas to reach a confining pressure of 300 psi and maintained at a constant temperature of 25 °C. The fluid was continuously stirred at 300 rpm until equilibrium was achieved between the gas and liquid phases. A drop in pressure within the cell serves as an indicator that the fluid has absorbed the maximum amount of CO_2_ under the given conditions.^34^ Once equilibrium was established, the final pressure was recorded. The quantity of CO_2_ absorbed (in moles) was then calculated using the real gas equation, as shown in eq 1.

1 $$n_{\mathrm{ab}}=\left(\frac{P_{1}}{Z_{1}}-\frac{P_{2}}{Z_{2}}\right)\cdot V/\mathrm{RT}$$

where *n*_ab_ denotes the number of moles of CO_2_ absorbed, *P*_1_ and *P*_2_ represent the initial and equilibrium pressures (in Pascals), *Z*_1_ and *Z*_2_ are the respective compressibility factors of CO_2_ at these pressures, *V* is the volume of the cell, *R* is the real gas constant (8.314 J/mol·K), and *T* is the absolute temperature in Kelvin. This method ensures the accurate quantification of gas absorption under controlled thermodynamic conditions.

#### 2.2.5 High-Pressure Interfacial Tension Measurement

The IFT was measured between the solutions and crude oil using a high-pressure tensiometer manufactured by DCAM Engineering, Ahmedabad, which employs the pendant/rising drop method at a confining pressure of 300 psi and a temperature of 25 °C. Before starting the experiment, the HPHT cell was completely vacuumed, and CO_2_ was added to maintain its confined pressure. The prepared carbonated slug was transferred to the cell, and the IFT was calculated under high-pressure conditions by incorporating an oil drop from the bottom.^35^

#### 2.2.6 Viscometry Analysis

Viscometric characterization of the prepared slugs was conducted using an Anton Paar MCR 302e rheometer equipped with a double-gap (DG35) geometry to ensure high sensitivity across a wide range of shear rates. The analysis was performed over a shear rate range of 1–1000 s^–1^ to evaluate the shear-dependent rheological behavior of the slugs. Two experimental conditions were employed: ambient (normal) and high pressure (carbonated). The slugs were tested under ambient conditions without any applied pressure. For high-pressure measurements, the rheometer cell was carefully filled with the slug formulation and then vacuumed to eliminate entrapped air, ensuring accurate measurement results. The carbonated slug was generated in situ within the double-gap geometry by applying a confining pressure of 300 psi, followed by a preshear at 100 s^–1^, until pressure equilibrium was achieved. Rheological tests were then performed under controlled pressurized conditions to assess the influence of carbonation on the flow behavior.^36^ This methodology enabled a comprehensive comparison of rheological responses under both normal and carbonated environments, thereby simulating realistic operational conditions.^37^

#### 2.2.7 Core Flooding

Core flooding experiments were conducted to assess the efficacy of the optimized slug at varying concentrations for enhanced oil recovery. The experimental setup shown in Figure 3 was designed and engineered by DCAM Engineering in Ahmedabad and consisted of a core holder, dual accumulators, and a high-precision syringe pump, all of which were designed to maintain a constant flow rate throughout the entire flooding process, at an ambient room temperature (24 ± 0.5 °C). Before the experiments, the sandstone core samples were rigorously cleaned with analytical-grade toluene and methanol to remove residual hydrocarbons and contaminants.^38^ The cores were subsequently dried in an oven at 120 °C for 24 h to ensure complete removal of moisture. Their petrophysical properties, including porosity and permeability, were quantified and are presented in Table 1.^6^ The core holder was subjected to a confining pressure of approximately 3,000 psi, complemented by a back pressure of 250 psi, to simulate the reservoir conditions accurately. After saturating the cores with crude oil to establish the initial oil saturation, a 24 h stabilization period was implemented. The tertiary recovery phase commenced with the injection of water, followed by an optimized slug pore volume (PV) of 0.5, and concluded with the injection of chase water at a consistent flow rate of 0.5 mL/min.

**Figure 3:**
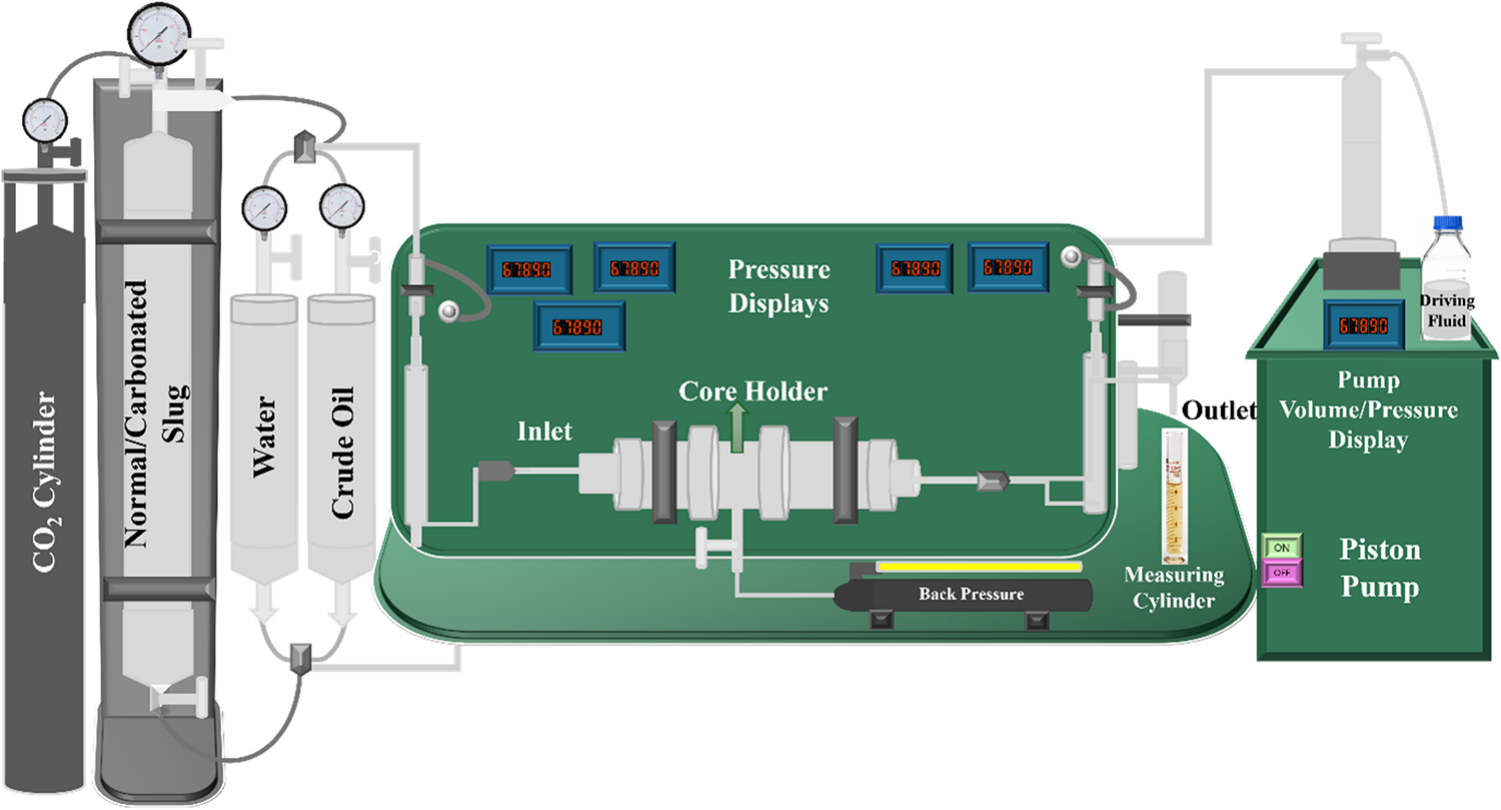
Schematic diagram for the core flooding experiment.

**Table 1:** Solid (Core) and Liquid (Crude Oil) Properties^6^

core DATA		crude oil DATA	
name	bandera gray	density (g/cm^3^)	0.94
diameter (cm)	3.81	API gravity (°)	18.58
length (cm)	6.51	pour point (°C)	8
bulk volume (cc or mL)	74.25	saturates (% w/w)	54.10
pore volume (cc or mL)	14.11	aromatics (% w/w)	29.28
porosity (%)	19	resins (% w/w)	7.40
gas permeability (mD)	14	asphaltenes (% w/w)	9.22

## 3 Results and Discussion

This section discusses the characterization of synthesized SL and its potential applications in enhanced oil recovery.

### 3.1 Sophorolipid Characterization

#### 3.1.1 FTIR Analysis

The FTIR spectrum of the biosurfactant revealed typical glycolipid functional groups (Figure 4). A broad band at 3400 cm^–1^ indicates −O–H stretching vibrations in the sugar and hydroxyl groups. Peaks at 2920 and 2850 cm^–1^ result from −C–H stretching of methylene and methyl groups, confirming the presence of multiple −C–H bonds throughout the structure.^40,41^ A sharp peak at 1730 cm^–1^ represents −C=O stretching, characteristic of both ester and carboxylic acid groups.^41^ This spectroscopic analysis reveals that the −O–H bond bends between 1450 and 1600 cm^–1^, confirming that carboxylic acids are significant contributors to the interfacial film. The band around 1100–1000 cm^–1^ corresponds to the −C–O stretching vibrations of secondary alcohols linked to the sugar, proving that the compound is a glycolipid biosurfactant, and its headgroup joins to other molecules by glycosidic bonds (Table 2).^42^

**Figure 4:**
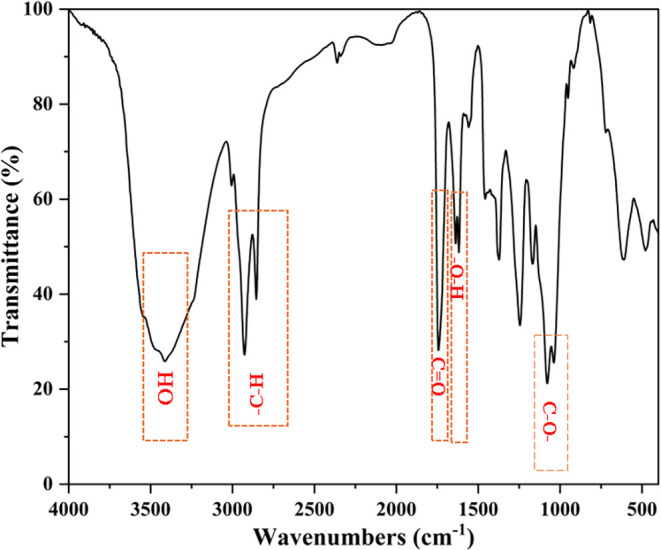
FTIR analysis.

**Table 2:** FTIR (Molecular Representation of SL)

wavenumber (cm^–1^)	functional group	vibration type	structural assignment
∼3400	–O–H (hydroxyl)	stretching	hydroxyl groups in sophorose and carboxylic acid
2920 and 2850	–C–H (aliphatic −CH_2_, – CH_3_)	asymmetric and symmetric stretching	long-chain fatty acid hydrocarbon tail
∼1730	–C=O (carbonyl)	stretching	ester and/or carboxylic acid group (lipid–sugar linkage)
1600–1450	–O–H (carboxylic acid)	bending	free carboxylic acid moieties
1100–1000	–C–O (alcohol/glycosidic)	stretching	secondary alcohols in the sophorose sugar ring

#### 3.1.2 NMR Analysis

^13^C NMR spectroscopy provides structural information regarding SL-based biosurfactants. The multiple peaks in the spectrum indicate that the molecule contains various carbon environments. Figure 5 demonstrates the NMR spectra. A distinct peak in the range of ∼172–174 ppm indicated ester or carboxylic carbonyl (−C=O) groups, indicating ester bonds between the fatty acid tail and sophorose sugar in the molecule. Oxygen makes the signals less shielded.^43^ In the range of 120 to 140 ppm, the peaks are sharp, indicating the presence of unsaturated fats and small amounts of aromatic matter, which gives the surfactant its ability to mix with water and oil. The existence of a strong peak at ∼80 ppm on carbon-1 reveals a glycosidic linkage, and the signals at ∼60–75 ppm on other carbons in the sugar ring prove that there are many hydroxyl groups in the sugar molecule. Peaks in the ∼38 ppm region are from the carbons near the carbonyl, and the ranges from ∼14–35 ppm show carbons from the fatty acid chains (Table 3).^44^

**Figure 5:**
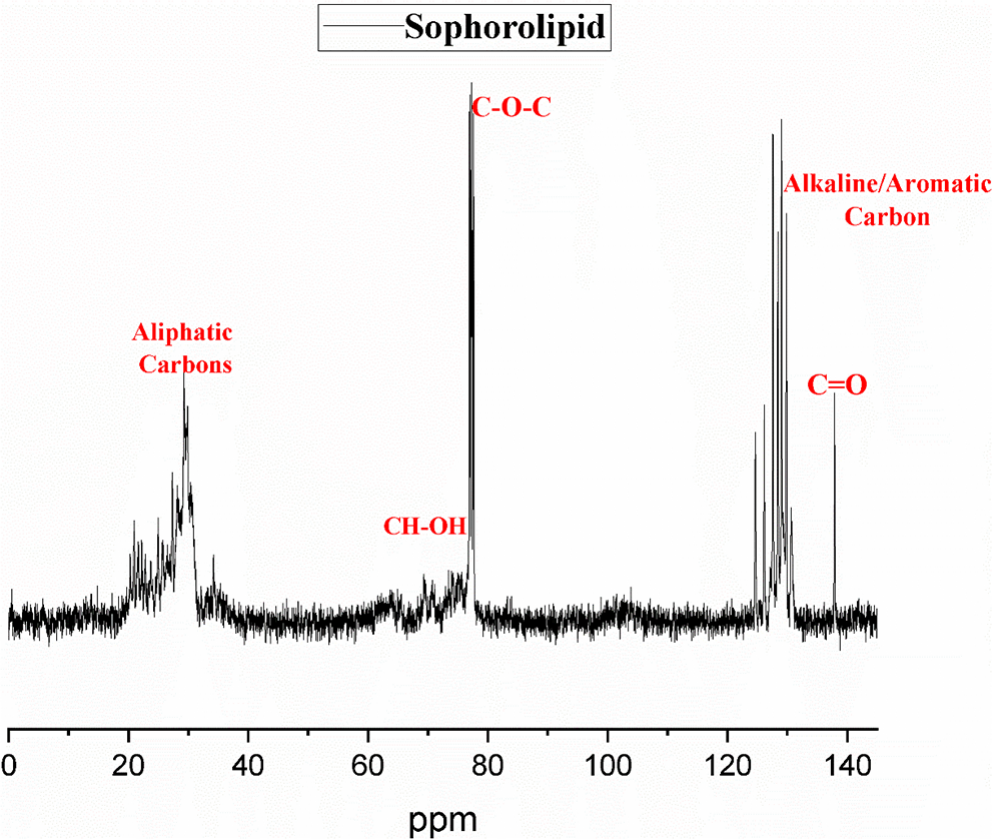
NMR spectra of sophorolipid.

**Table 3:** NMR Analysis of SL

ppm	peak	chemical bonds	structural component
14–35	aliphatic carbons (CH_3_, CH_2_)	methyl and methylene group	long hydrocarbon fatty acid chain
38	α-carbon to ester/acid group	CH_2_–C=O (deshielded due to carbonyl)	fatty acid linkage (α-carbon)
60–75	sugar ring carbons (CH–OH)	oxygenated secondary carbons	sophorose moiety (glucose-like)
80	anomeric carbon (C–O–C)	highly deshielded due to glycosidic linkage	sophorose glycosidic bond
172–174	ester or carboxylic carbonyl (C=O)	carbon of ester/acid group	fatty acid/sophorose ester group
120–140	alkaline/aromatic like carbons	possibly unsaturated fatty acids or impurities	fatty acid tail with unsaturation

### 3.2 Critical Micelle Concentration (CMC) and IFT Determination

The critical micelle concentration (CMC), which represents the threshold concentration of surfactant for micelle formation, was determined through surface tension measurements using the Wilhelmy plate technique. SL exhibited a CMC of 250 ppm, indicating significant surface tension reduction and micelle formation driven by its amphiphilic structure comprising hydrophilic sophorose heads and hydrophobic fatty acid tails.^45^ SLs offer advantages such as biodegradability, low toxicity, and renewable production. The ability to significantly reduce the surface tension from 72 to 31 mN/m highlights their EOR potential.^3^

Interfacial tension measurements demonstrated the surface-active properties of the biosurfactant at various concentrations (0–300 ppm). The control sample (0 ppm) exhibited the highest interfacial tension, at 14.62 ± 0.8 mN/m, representing the baseline tension between the two phases in the absence of any surfactant. The reduction in interfacial tension was observed upon biosurfactant addition, with the most significant decrease occurring at 50 ppm (5.72 ± 0.3 mN/m), representing a 61% reduction compared to the control.^46^ This steep initial drop indicates a high surface activity and efficient partitioning of the biosurfactant molecules at the interface. Further increases in SL concentration continued to reduce interfacial tension, reaching a minimum value of 2.2 ± 0.1 mN/m at 250 ppm, an 85% reduction from the control. This represents the critical micelle concentration (CMC) region, where the maximum surface activity is achieved. Whereas, at 300 ppm, interfacial tension showed a marginal increase to 3.12 ± 0.2 mN/m, suggesting that the CMC has been exceeded. Beyond CMC, additional SL molecules form micelles in the bulk phase, rather than contributing to further reduction of interfacial tension, as shown in Figure 6. The biosurfactant demonstrated excellent surface activity, achieving interfacial tension values below 3 mN/m, which are considered highly effective for EOR applications. The low error bars throughout the concentration range indicated good reproducibility of the measurements and consistent biosurfactant performance.^22^

**Figure 6:**
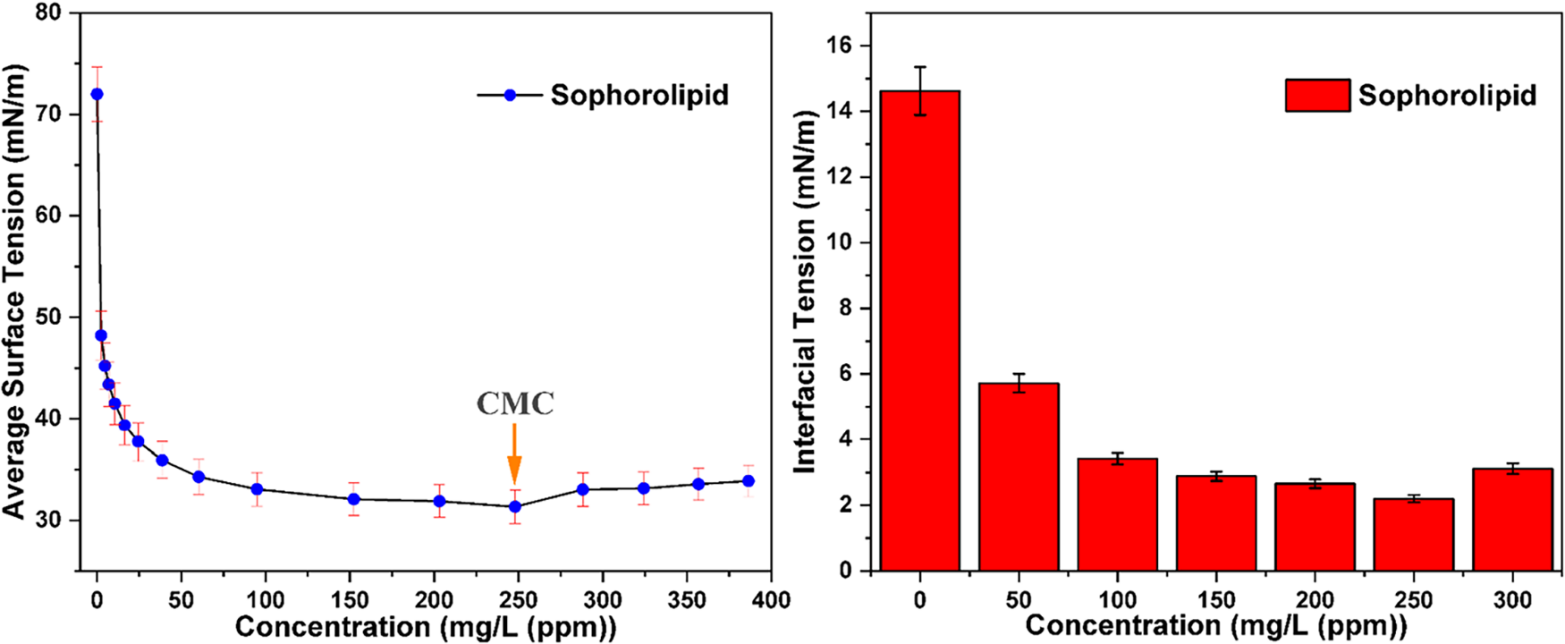
CMC (1) and IFT (2) of sophorolipid.

### 3.3 Wettability Analysis

The contact angle analysis demonstrates a clear transition in surface wettability upon treatment with SLs. Initially, the untreated surface at 0 ppm displayed a high contact angle of 106°, indicative of strong hydrophobicity, where the water droplets maintained a spherical shape and did not spread, highlighting the poor surface wettability. However, with the gradual addition of SL, ranging from 50 to 250 ppm, a consistent decrease in contact angle was observed, confirming the enhanced wettability of the surface.^47^ At 50 ppm, the contact angle dropped notably to 87°, indicating the initial adsorption of SL molecules and a reduction in the surface tension. As the concentration increased to 100 and 150 ppm, the contact angles further decreased to 81° and 74°, respectively, indicating a denser surfactant layer on the surface and a corresponding shift toward a hydrophilic character. At higher concentrations of 200 and 250 ppm, the contact angles decreased significantly to 54 and 48°, respectively, indicating that the surface became strongly hydrophilic, as shown in Figure 7. This behavior is attributed to the amphiphilic structure of the biosurfactant, which comprises a hydrophilic sugar moiety and a hydrophobic fatty acid chain that enables the molecules to orient at the interface with their hydrophilic ends exposed outward. This configuration reduces the surface energy, allowing water to spread more easily. Beyond a certain concentration, which corresponds to the critical micelle concentration (CMC), excess SL molecules aggregate into micelles, ensuring saturated and stable surface modification that maintains low contact angles.^48^ In conclusion, the gradual decline in the contact angle with an increasing SL concentration confirms its efficacy in modifying surfaces from hydrophobic to hydrophilic. Such surface modification through biosurfactant application holds promise for various industrial and environmental applications, with optimal performance observed at a concentration of 250 ppm.

**Figure 7:**
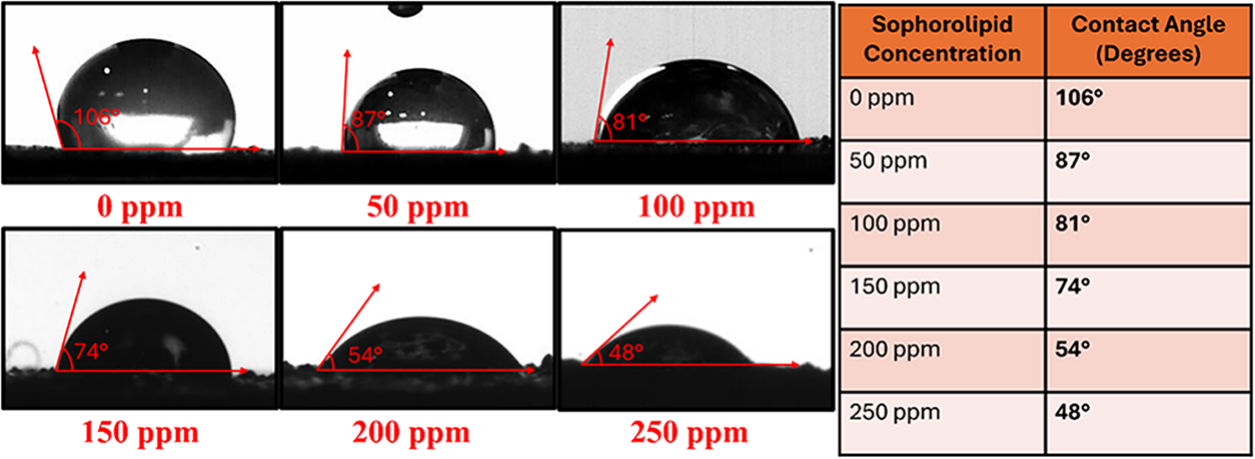
Contact angle versus time for different concentrations of sophorolipid.

The time-resolved contact angle analysis presented here provides a comparative evaluation of the dynamic wettability behavior of various formulations, including SL, polyacrylamide (PAM), and their combinations. The images and corresponding graphs reveal the evolution of the contact angle over 10 min for each sample, offering insights into the wetting efficiencies and interfacial interactions. At the initial time point (0 min), all samples show relatively high contact angles, with pure PAM solutions (500, 1000, and 1500 ppm) maintaining angles in the intermediate-wet range (above 75°), and SL-based systems, particularly those with 250 ppm of SL alone, exhibiting lower angles indicative of water-wet behavior.^49^ As time progressed, a marked reduction in the contact angle was observed for systems containing SL. Notably, 250 ppm of SL alone showed a rapid and steady decline from ∼48 to ∼25° in 600 s, confirming its high surface activity and ability to strongly enhance wettability.

Pure PAM solutions, on the other hand, retained higher contact angles throughout the test period, with 500 ppm of PAM remaining nearly unchanged (∼75°). In contrast, 1000 and 1500 ppm PAM showed only slight decreases over time, confirming their poor wettability and intermediate-wet nature.^50^ This highlights that PAM alone is not sufficient to induce strong water-wet conditions, but when paired with biosurfactants, such as SL, their surface-altering potential significantly improves.

A synergistic effect was observed when SL was combined with PAM (250 ppm of SL + 500, 1000, and 1500 ppm of PAM). For instance, 250 ppm of SL + 500 ppm of PAM resulted in a significant drop in the contact angle to below 35° within the first few minutes, reaching values comparable to or even lower than those of SL alone, suggesting accelerated wetting due to enhanced interfacial adsorption. In contrast, higher PAM concentrations (1000 and 1500 ppm), in combination with SL, initially moderated the reduction in contact angle, but eventually achieved improved wettability compared to PAM alone, as shown in Figure 8. These results indicate that although PAM is less surface-active on its own, its interaction with SL possibly improves the mobility or orientation of the surfactant at the interface, particularly at optimized concentrations. The contact angle data indicate that SL substantially enhances the surface wettability, especially when combined with PAM at optimal concentrations. The trend toward lower contact angles over time signifies increased surface wetting and lower interfacial tension. This time-dependent transformation from intermediate-wet to water-wet states is particularly crucial in applications such as enhanced oil recovery, soil remediation, or any interface-sensitive process where quick and efficient wettability alteration is desired.

**Figure 8:**
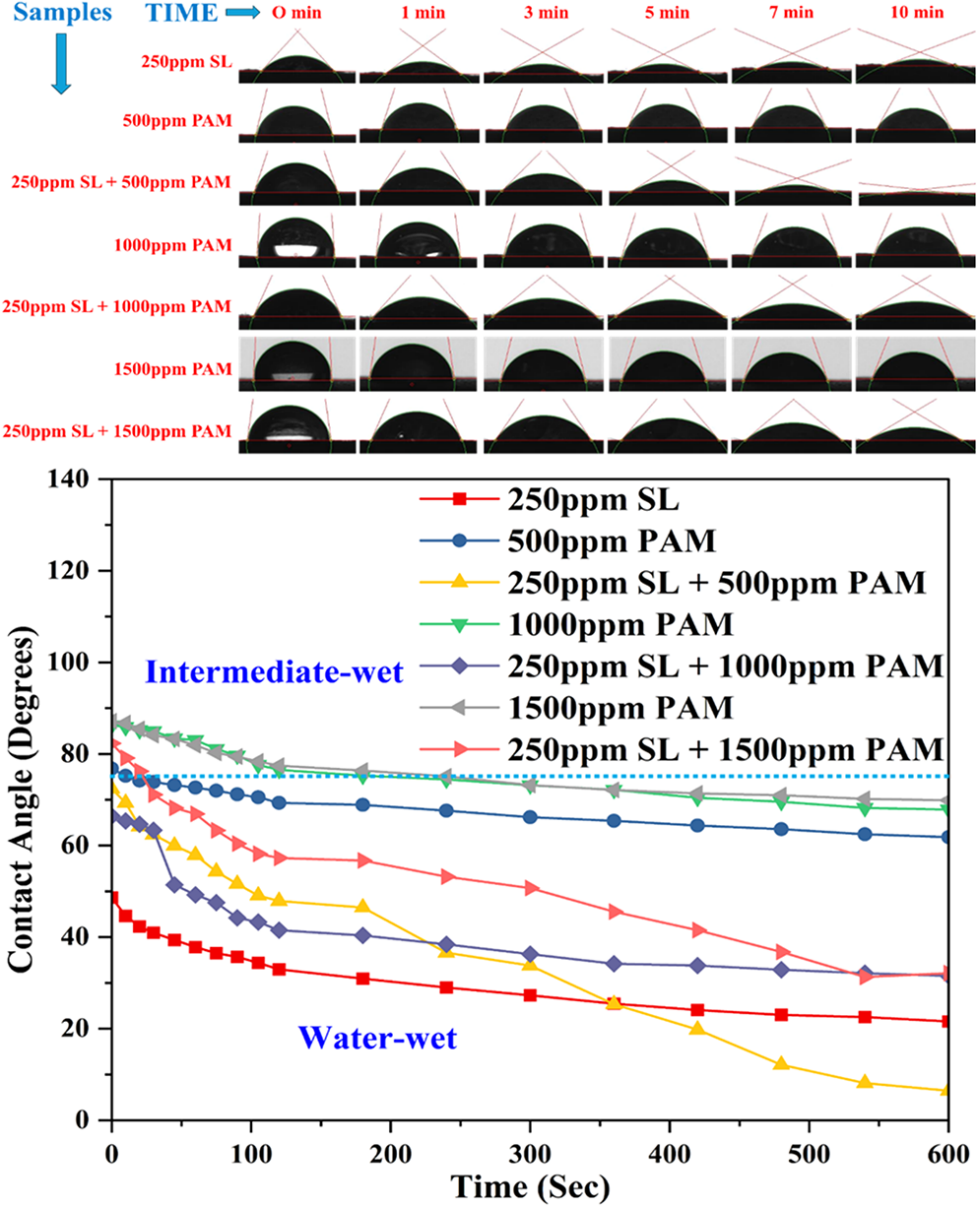
Contact angle images/values with the corresponding times of different solutions.

### 3.4 CO_2_ Absorption

The CO_2_ absorption capacity measurements across different formulations reveal critical insights into the synergistic behavior of SL and polyacrylamide (PAM) in facilitating CO_2_ solubilization through distinct but complementary mechanisms.^51^ The baseline absorption in deionized water was recorded at 0.46515 mol/kg, reflecting the limited gas solubility governed by Henry’s law and the inherently low CO_2_-water interfacial area in the absence of surface-active agents. The introduction of 250 ppm of SL alone markedly enhanced CO_2_ uptake to 0.72467 mol/kg (56% increase), demonstrating the efficiency of biosurfactant micelles in augmenting gas–liquid interactions through multiple mechanistic pathways.^52^ The amphiphilic nature of SL enables spontaneous self-assembly into micellar structures above the critical micelle concentration, creating hydrophobic microenvironments within the micellar core that preferentially solubilizes CO_2_ molecules through favorable van der Waals interactions and reduced solvation energy requirements. Additionally, SL molecules adsorbed at gas–liquid interfaces significantly increase the effective interfacial area available for CO_2_ mass transfer. In contrast, the dynamic nature of micellar equilibrium facilitates continuous CO_2_ exchange between the bulk solution and micellar phases, effectively increasing the apparent solubility coefficient beyond the thermodynamic predictions for simple physical dissolution.^52^

PAM at increasing concentrations (500, 1000, and 1500 ppm) showed marginal improvements in CO_2_ absorption (0.522 to 0.57861 mol/kg), representing modest 12–24% enhancements over baseline water absorption, which is primarily attributable to rheological modifications rather than chemical interactions. The mechanism involves increased solution viscosity and molecular entanglements created by PAM’s high-molecular-weight polymer chains, which reduce the CO_2_ bubble rise velocity and extend the gas–liquid contact time, thereby improving mass transfer efficiency through prolonged residence time rather than enhanced thermodynamic solubility.^53^ The polymer network also creates temporary CO_2_ entrapment sites within the three-dimensional macromolecular structure, where gas molecules become physically constrained by polymer chain entanglements, leading to an apparent absorption enhancement through kinetic rather than equilibrium effects.^1,54^ However, PAM’s predominantly hydrophilic character limits its ability to provide favorable CO_2_ interaction sites, explaining its modest improvements compared to surfactant-based systems. The dual-component systems containing PAM and 250 ppm of SL exhibited remarkable synergistic effects, exceeding the additive contributions of the individual components. Specifically, 500PAM + 250SL and 1000PAM + 250SL reached 0.747 and 0.79155 mol/kg, respectively. At the same time, the 1500PAM + 250SL formulation achieved the highest CO_2_ absorption, at 0.82486 mol/kg (a 77% enhancement over the baseline). This synergistic behavior operates through multiple interconnected mechanisms. First, SL micellar aggregates facilitate enhanced CO_2_ solubilization by increasing the availability of a hydrophobic microenvironment, where polymer–surfactant interactions modify the micellar structure and stability, potentially increasing micellar size and creating larger hydrophobic cores with greater CO_2_ accommodation capacity.^55^ Second, PAM chains interact with SL molecules through hydrogen bonding between amide groups and sophorose hydroxyl groups, forming polymer–surfactant complexes that exhibit modified interfacial properties and enhanced surface activity compared with free SL molecules. Third, the polymer network stabilizes the micellar architecture by reducing micellar dynamics and preventing coalescence, thereby maintaining an optimal micellar size distribution and prolonging the CO_2_ residence time within the hydrophobic domains. Fourth, PAM’s viscosity enhancement works synergistically with SL’s interfacial activity, reducing mass transfer limitations and creating a more stable gas–liquid dispersion with an increased interfacial area.^52^ The optimal performance at 1500PAM + 250SL suggests a critical balance, where the maximum polymer–surfactant interaction occurs without steric hindrance or micelle disruption, indicating that higher PAM concentrations provide sufficient polymer chain density to fully stabilize the SL micellar network while maintaining accessibility for CO_2_ solubilization, as shown in Figure 9.

**Figure 9:**
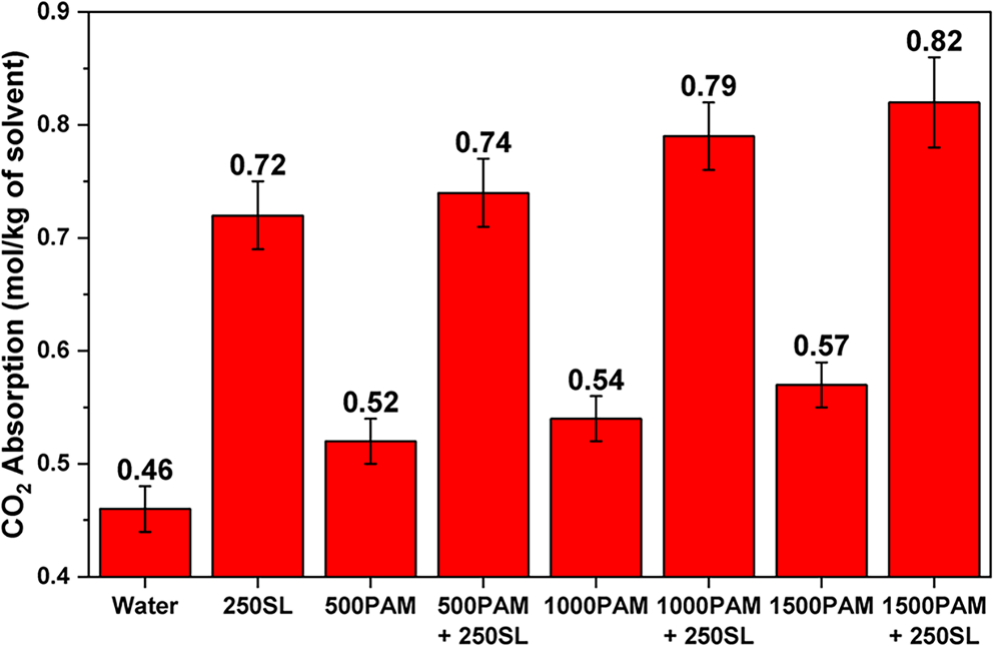
CO_2_ absorption of different solutions.

These results demonstrate that the enhanced CO_2_ absorption in biosurfactant–polymer hybrid systems results from a combination of thermodynamic enhancements (increased apparent solubility through micellar solubilization), kinetic improvements (extended contact time through viscosity modification), and interfacial optimization (increased surface area and reduced interfacial tension), confirming the effectiveness of these formulations as potential candidates for eco-friendly and high-efficiency CO_2_ capture processes under ambient conditions with applications in carbon sequestration and enhanced oil recovery.^47,54^

### 3.5 High-Pressure Interfacial Tension Measurement

Pure water established baseline interfacial tension values of 15.12 mN/m under ambient conditions and 12.2 mN/m under elevated CO_2_ pressure, with the pressure-induced reduction reflecting CO_2_ dissolution into the aqueous phase that decreases water density and modifies hydrogen bonding networks, consequently lowering the surface energy by 19%. The introduction of 250 ppm of SL resulted in dramatic interfacial tension reductions to 2.2 mN/m (ambient) and 2.18 mN/m (pressurized conditions), representing an 85% decrease from baseline values due to SL’s unique molecular architecture comprising a hydrophilic sophorose disaccharide headgroup connected to hydrophobic fatty acid tails through glycosidic linkages.^56^ At the gas–liquid interface, SL molecules undergo spontaneous adsorption driven by thermodynamic favorability, with the sophorose moiety anchoring in the aqueous phase through extensive hydrogen bonding with water molecules. At the same time, the fatty acid chains orient toward the gas phase, minimizing unfavorable hydrophobic–hydrophilic interactions and creating a tightly packed monolayer that significantly reduces the interfacial free energy. The minimal pressure dependence (2.2 vs 2.18 mN/m) indicates robust interfacial stability, suggesting that CO_2_-enriched conditions do not disrupt the established SL molecular arrangement due to strong intermolecular interactions within the adsorbed layer.^54^

The PAM solutions (500–1500 ppm) exhibited consistently high IFT values, ranging from 52.36 to 43.78 mN/m under ambient conditions and from 48.68 to 36.85 mN/m under pressure, with these elevated values exceeding even those of pure water due to PAM’s predominantly hydrophilic backbone, which lacks significant surface-active functionality. PAM’s linear polymer chains contain amide groups that form extensive hydrogen bonds with water molecules, increasing bulk phase cohesion and elevating interfacial tension. Slight pressure-mediated reductions likely arise from CO_2_-induced conformational changes in polymer chains or the partial disruption of intermolecular associations, although these effects are insufficient to impart meaningful surface activity. The PAM + SL combinations demonstrated remarkable synergistic behavior, achieving IFT values of 6.73 mN/m (500PAM + 250SL), 6.52 mN/m (1000PAM + 250SL), and 6.24 mN/m (1500PAM + 250SL) under ambient conditions, with similar performance maintained under CO_2_ pressure through multiple synergistic mechanisms, including polymer–surfactant complex formation via hydrogen bonding between amide groups and sophorose hydroxyl groups, interfacial rheological modification where PAM increases the viscoelasticity of the interfacial film to stabilize SL arrangements and prevent desorption, cooperative adsorption where PAM chains facilitate SL transport to the interface through associative interactions, and pressure-enhanced interactions where dissolved CO_2_ may plasticize PAM chains to optimize SL-PAM cooperative arrangements at the interface.

The optimal performance at 1500PAM + 250SL (6.24 mN/m ambient and 6.18 mN/m pressurized) indicates a critical balance between polymer chain density and surfactant availability, where PAM provides sufficient interfacial stabilization without sterically hindering SL adsorption. At the same time, the SL concentration remains adequate for effective surface coverage despite polymer interactions. These mechanistic insights validate the potential of the system for CO_2_-enhanced oil recovery and carbon sequestration applications, with the pressure-tolerant performance indicating stability under reservoir conditions and the synergistic behavior suggesting opportunities for further optimization through molecular weight tuning of PAM to optimize polymer–surfactant interaction strength, SL structural modification to engineer SL congeners with enhanced CO_2_ compatibility, and concentration ratio optimization to maximize synergistic effects while maintaining economic viability, as shown in Figure 10. The results demonstrate that while SL drives interfacial activity through classical surfactant mechanisms involving amphiphilic molecular orientation and interfacial energy reduction, PAM’s role as a rheological modifier creates synergistic interfacial films with superior performance compared to individual components, particularly under the challenging conditions relevant to subsurface applications where the combination achieves ultralow interfacial tensions essential for enhanced oil recovery efficiency and CO_2_ sequestration processes.^57^

**Figure 10:**
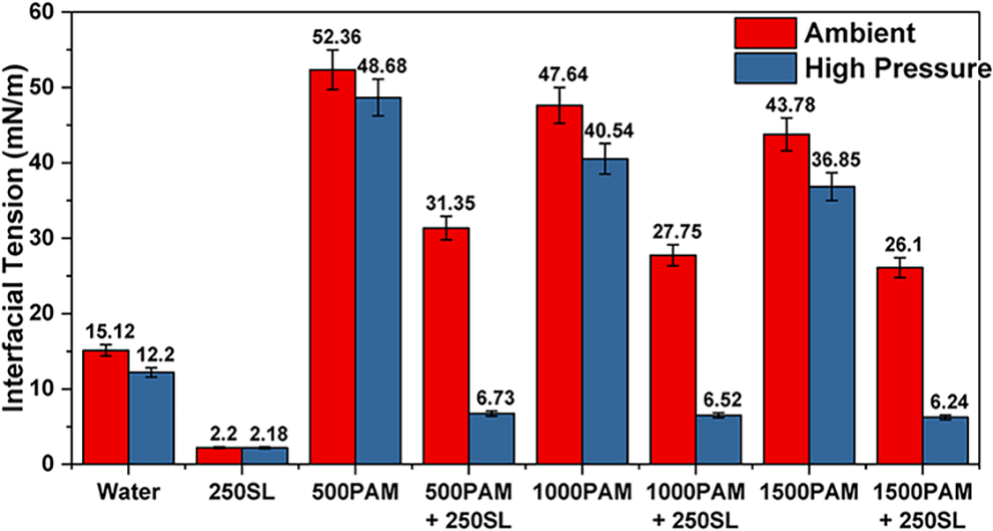
High-pressure interfacial tension results.

### 3.6 Viscosity Analysis

Rheology plays a crucial role in evaluating the flow behavior of biosurfactant–polymer formulations, particularly for applications such as enhanced oil recovery (EOR), where fluid viscosity has a direct impact on performance. The combination of SL and polyacrylamide (PAM) at varying concentrations was investigated to understand their viscoelastic properties under different shear rates. This analysis helps to reveal how molecular interactions respond to mechanical stress.^36^

Rheological analysis, illustrated in the graph, demonstrates the shear-thinning behavior of the biosurfactant–polymer (SL-PAM) systems at 25 °C. All three formulations, 250SL + 500P, 250SL + 1000P, and 250SL + 1500P, exhibited a pronounced decrease in viscosity with increasing shear rate, which is a typical characteristic of non-Newtonian, pseudoplastic fluids.^36,50^ Initially, the viscosity values were relatively high at low shear rates (∼1 s^–1^), indicating a strong intermolecular network and a structural resistance to flow. Among the tested combinations, the 250SL + 1500P formulation (blue triangles) consistently exhibited the highest viscosity across all shear rates, followed by the 250SL + 1000P (red circles) and 250SL + 500P (black squares) formulations (Figure 11). This trend indicates that increasing the polyacrylamide (PAM) concentration enhances the system’s resistance to shear, likely due to increased polymer entanglement and interaction with the biosurfactant molecules.^36^

**Figure 11:**
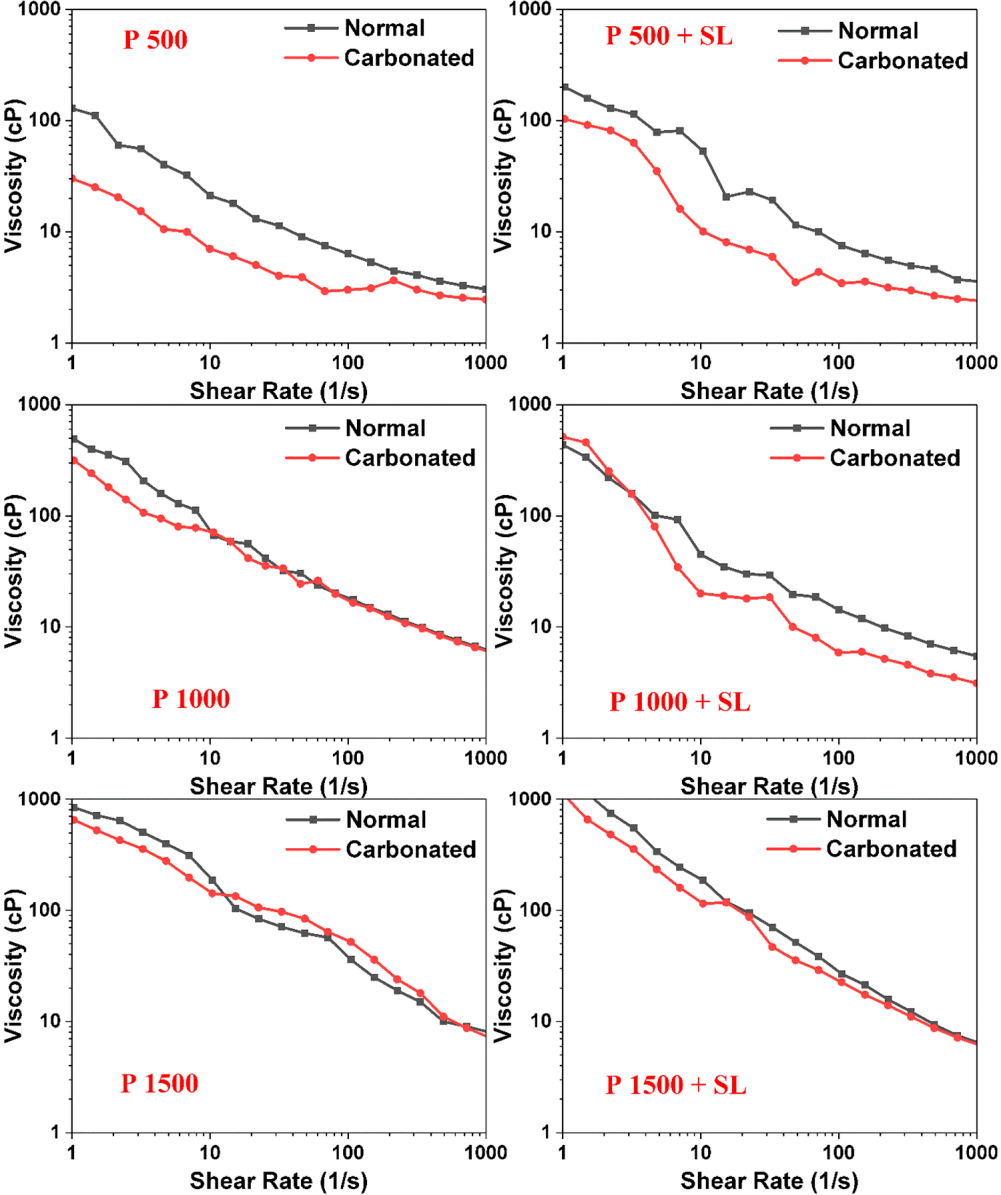
Viscosity vs shear rate graphs.

As the shear rate increased, the viscosity declined steadily for all samples, indicating the alignment and disentanglement of the polymer chains under shear stress. At higher shear rates (∼1000 s^–1^), the viscosity values of all three systems converged, suggesting structural breakdown and minimal resistance to flow. The observed rheological profile is highly favorable for applications such as enhanced oil recovery (EOR),^39^ where fluids must maintain high viscosity at low flow rates for effective mobility control and exhibit reduced viscosity under high-shear conditions to facilitate injection. The synergistic effect between the SL biosurfactant and PAM enhances viscosity retention, especially at higher PAM concentrations, confirming the potential of the formulation in shear-sensitive processes.

#### 3.6.1 CO_2_ Effect on Viscosity

The viscosity–shear rate CO_2_ dissolution into the SL-polyacrylamide formulations significantly impacts rheological behavior across all tested concentrations, with all three systems (250SL + 500P, 250SL + 1000P, and 250SL + 1500P) exhibiting pronounced shear-thinning characteristics under carbonated conditions.^58^ The formulations demonstrated initial viscosities of approximately 600, 850, and 950 mPa·s at low shear rates (1 s^–1^), systematically decreasing to converged values of 8–12 mPa·s at high shear rates (1000 s^–1^), indicating CO_2_’s plasticizing effect on the polymer–surfactant network.^36^ CO_2_ molecules preferentially dissolve into SL micellar domains and interact with polyacrylamide chains, disrupting intermolecular associations and partially disintegrating polymer–surfactant bridges, resulting in an overall reduction in viscosity compared to noncarbonated systems. The 250SL + 1500P formulation exhibited the highest initial viscosity and most pronounced shear-thinning behavior, demonstrating the maximum CO_2_ accommodation capacity within its three-dimensional network structure while maintaining the most stable viscosity profile across the tested shear range.^6^ The convergence of all formulations to similar viscosity values at high shear rates suggests that CO_2_-induced modifications become less significant under high-shear conditions where mechanical forces dominate, confirming CO_2_’s ability to modify the spatial arrangement and fluidity of the polymer–surfactant network with the reduction extent correlating directly to polymer concentration, making the highest concentration system optimal for enhanced oil recovery applications requiring controlled viscosity under varying flow conditions in CO_2_-rich environments.

### 3.7 Core Flooding Experiments

Core flooding experiments were conducted to assess the oil recovery efficiencies of various concentrations of SL slugs, both in isolation and in conjunction with a polymer (PAM), under carbonated conditions at an ambient room temperature (24 ± 0.5 °C). We have conducted the core flooding experiments at ambient temperature due to limitation, although the produced biosurfactant was quite stable at wide range of salinity, pH, and temperatures up to 100 °C.^20^ Table 4 summarizes the core properties, saturation states, and oil recovery results for each flooding scenario.^59^

**Table 4:** Oil Recovery at Varying Concentrations

	*K* (mD)		saturation (%)						
chemical composition of slug	*K*_w_ at *S*_w_ = 1	*K*_o_ at soi	soi	swi	sor	secondary oil recovery (% OOIP)	additional oil recovery (% OOIP)	Δ*P* (psi)	cumulative oil recovery (% OOIP)
100SL	1.59	0.06	77.97	22.03	45.72	41.36	2.74	1465	44.10
250SL	1.54	0.05	81.52	18.48	47.49	41.73	9.57	1765	51.30
500SL	1.56	0.05	79.35	20.65	43.59	44.10	3.62	1622	47.72
1500PAM	1.54	0.06	81.52	18.48	47.14	42.17	5.65	2047	47.83
C 1500PAM	1.59	0.06	80.00	20.00	47.85	41.30	13.48	2168	54.78
250SL + 1500PAM	1.59	0.06	78.24	21.76	42.53	45.45	15.46	2433	60.91
C 250SL + 1500PAM	1.50	0.04	85.06	14.94	44.66	47.50	24.17	2511	71.67

Initial flooding trials utilizing pure SL slugs at concentrations of 100, 250, and 500 ppm revealed distinct levels of effectiveness in enhancing oil recovery. Notably, the 250 ppm of SL slug achieved the highest cumulative oil recovery at 51.3% of the original oil in place (OOIP). This optimal performance was attributed to a favorable interfacial tension reduction and enhanced wettability alteration. The scenario exhibited a secondary recovery of 47.49% and an additional 9.57% during the tertiary phase, indicating the efficient mobilization of residual oil.

Conversely, lower 100 and higher 500 ppm of SL concentrations resulted in lower total recoveries of 44.1 and 47.72% OOIP, respectively. Although the 500 ppm condition recorded a marginally higher tertiary recovery (3.62%) compared to the 100 ppm condition (2.74%), the findings suggest that excessive surfactant concentrations may hinder efficiency due to potential surfactant aggregation or increased adsorption to the rock matrix.^60^

The optimal rise in differential pressure (Δ*P*) was observed at a concentration of 250 ppm of sophorolipid (SL), beyond which further increases in SL concentration led to a continued rise in pressure. Notably, the combination of 250 ppm of SL with 1500 ppm of PAM resulted in a significant pressure increase to 2433 units. The maximum differential pressure, however, was achieved with the carbonated formulation of 250 ppm of SL + 1500 ppm of PAM, reaching 2511 units, indicating enhanced flow resistance and superior plugging performance at this composition. To further optimize the recovery, the effective 250 ppm of SL slug was paired with 1500 ppm of PAM, a water-soluble polymer known to enhance sweep efficiency and improve mobility ratios. The combination of 250SL + 1500PAM significantly increased the cumulative oil recovery to 60.91% OOIP, with an additional 15.46% during the tertiary phase. This synergy illustrates the advantageous interplay between the surfactant and polymer in enhancing the displacement efficiency and reducing residual oil saturation.^6^

The most pronounced results were obtained when this optimized blend was used under carbonated conditions (C 250SL + 1500PAM) at an elevated pressure (3000 psi) in the presence of CO_2_. This scenario resulted in a remarkable cumulative oil recovery of 71.67% OOIP, with 24.17% attributed to tertiary recovery. The introduction of CO_2_ likely facilitated further reductions in crude oil viscosity, enhanced miscibility, and contributed to additional decreases in interfacial tension, which collectively improved the mobilization of entrapped oil.

The water permeability (*K*_w_) and oil permeability (*K*_o_) remained relatively stable across all experiments, underscoring the uniformity and integrity of the core samples. Notably, the lowest oil permeability (0.045 mD) was recorded during carbonated polymer–biosurfactant flooding,^61,62^ coinciding with the highest oil recovery, suggesting effective displacement of oil from pore spaces and increased flow resistance due to enhanced sweep coverage, which has been provided in Figure 12.

**Figure 12:**
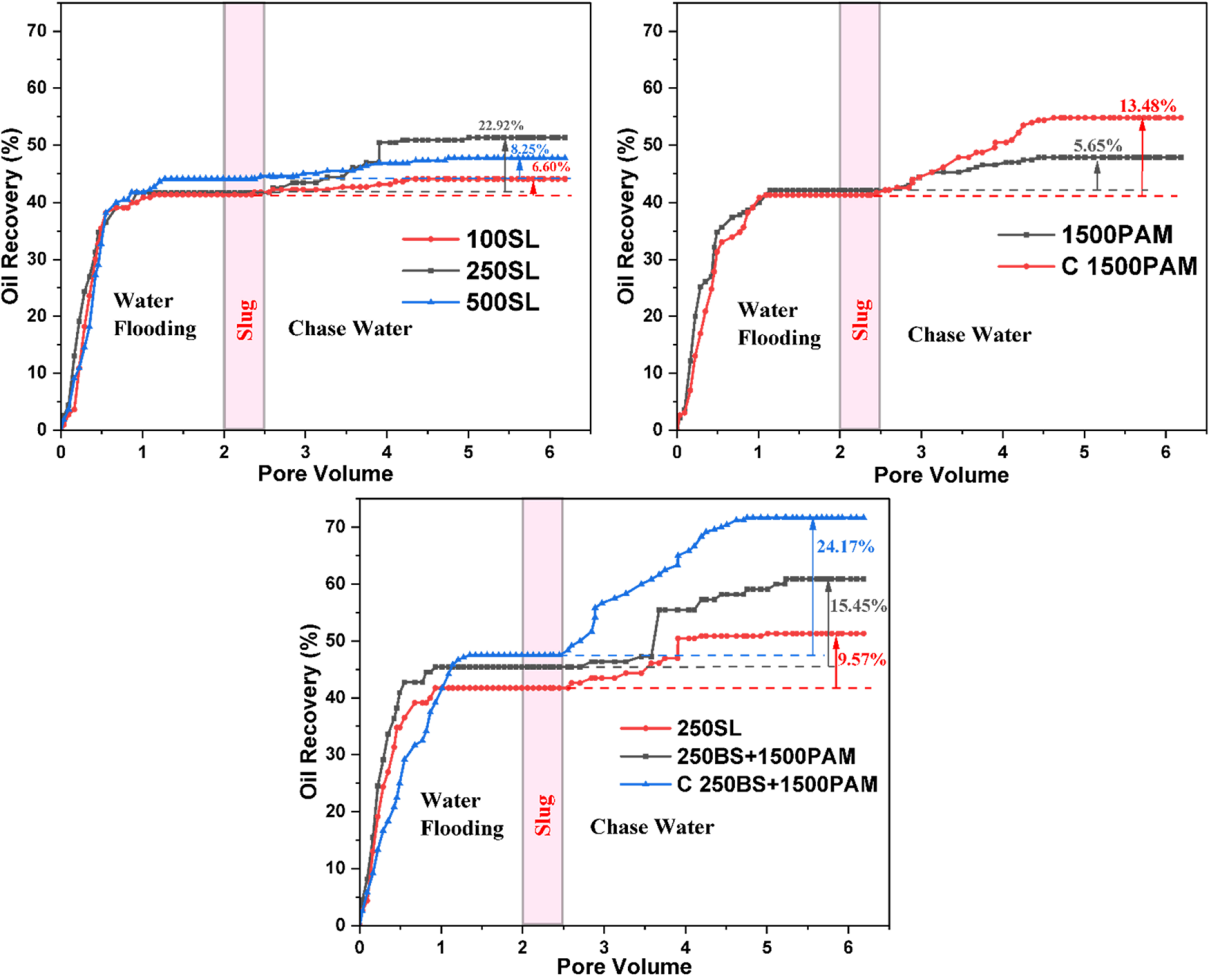
Oil recovery and differential pressure vs pore volume injected results of the core flooding experiments.

In summary, the results conclusively indicate the following:

1. The 250 ppm biosurfactant concentration is the most effective for standalone recovery enhancement.
2. Combining the surfactant with PAM significantly improved tertiary recovery rates.
3. Carbonated polymer–biosurfactant flooding resulted in the highest oil recovery, validating its applicability in advanced enhanced oil recovery (EOR) strategies.

### 3.8 Techno-Economic Analysis and Application Prospects

The economic viability of SL-based EOR technologies represents a critical factor for the field-scale implementation. Current production costs for sophorolipids range from USD 5–8 per kg when utilizing renewable feedstocks such as waste cooking oil and lignocellulosic hydrolysates, compared to USD 15–25 per kg for synthetic surfactants and USD 10–15 per kg for rhamnolipid biosurfactants produced by pathogenic organisms.^63,64^ The cost advantage of SL is further enhanced by its significantly lower critical micelle concentration (250 ppm) compared to conventional surfactants (500–2000 ppm), reducing the required injection volumes by 50–75%. Recent techno-economic assessments indicate that integrated biorefineries producing SL alongside other value-added products could reduce production costs to USD 3–5 per kg, making biosurfactant EOR economically attractive, even in mature fields with marginal production rates. The application prospects are particularly promising in environmentally sensitive regions where regulatory frameworks favor biodegradable chemicals,^65^ offshore operations where toxicity concerns are paramount, and CO_2_-EOR projects seeking carbon credits through demonstrated sequestration benefits. The dual functionality of SL systems in enhancing oil recovery while facilitating carbon storage positions them as strategic technologies for the energy transition, potentially qualifying for carbon offset mechanisms that could provide additional revenue streams of USD 20–40 per tonne of CO_2_ sequestered.^37^

## 4 Conclusions

This comprehensive study highlights SL-based biosurfactants as highly promising candidates for sustainable enhanced oil recovery (EOR) applications, demonstrating superior performance characteristics while addressing critical environmental concerns associated with conventional synthetic surfactants. Systematic characterization confirmed the unique glycolipid structure of SLs produced by *C. bombicola*, which features optimal amphiphilic properties essential for effective EOR applications. The excellent surface-active properties demonstrated by SLs, including an interfacial tension reduction (85% decrease to 2.2 mN/m) and efficient wettability alteration from oil-wet to water-wet conditions, underscore their technical efficacy in mobilizing trapped oil. The optimal critical micelle concentration of 250 ppm ensures economic viability for large-scale field applications, while maintaining exceptional performance standards. The synergistic integration of SLs with polyacrylamide resulted in significant performance enhancements, particularly in terms of rheological modification and CO_2_ absorption capacity. The 250 ppm of SL + 1500 ppm PAM formulation achieved 77% higher CO_2_ absorption than water alone, demonstrating its dual functionality for enhanced oil recovery and carbon sequestration applications. This synergy simultaneously addresses both production efficiency and climate change mitigation goals. Core flooding experiments provided compelling evidence of progressive recovery improvements, from 51.31% with standalone SLs to an exceptional 71.67% with carbonated SL–polymer systems. The 24.17% tertiary recovery achieved under carbonated conditions represents a significant advancement in EOR technology, validating its practical applicability under realistic reservoir conditions. The system maintains ultralow interfacial tension under high-pressure CO_2_, confirming technical viability. The pressure-tolerant performance and chemical stability under harsh reservoir conditions (high temperatures, pressures, and salinities) confirm the robustness of the SL-based systems for subsurface applications. The low interfacial tension maintained under CO_2_-enriched environments (2.18 mN/m) ensures consistent performance throughout the recovery process. Furthermore, the environmental advantages of SLs, including biodegradability, low toxicity, and renewable production pathways, have positioned them as cornerstone technologies for sustainable petroleum production. The ability to utilize renewable carbon sources and industrial waste streams for production further enhances their economic and environmental profiles. SL-based systems use agro-industrial waste, cutting production costs and addressing waste management. The safe production organism (*C. bombicola*) avoids biosafety concerns. Enhanced CO_2_ absorption improves the efficiency and enables carbon sequestration. For future work, studies should optimize for reservoir conditions, test long-term stability, assess integration with current infrastructure, and analyze environmental impacts. This study demonstrates that SL-based carbonated water flooding represents a paradigm shift toward environmentally responsible EOR operations, successfully balancing petroleum production efficiency with ecological integrity. These promising results warrant field-scale implementation studies and continued optimization for commercial deployment in next-generation sustainable oil recovery operations. SL-based carbonated water flooding offers a sustainable, cost-effective EOR option that supports both production and climate goals.

## Abbreviations

EOR: enhanced oil recovery
SL: sophorolipid
PAM: polyacrylamide
CMC: critical micelle concentration
IFT: interfacial tension
ST: surface tension
**CO**_**2**_: carbon dioxide
MEOR: microbial enhanced oil recovery
FTIR: Fourier transform infrared (spectroscopy)
NMR: nuclear magnetic resonance
PNF: purified natural fractions
HPHT: high pressure high temperature
DI: deionized (water)
KBr: potassium bromide
**D**_**2**_ **O**: deuterium oxide
VVM: volume of air per volume of liquid per minute
OOIP: original oil in place
PV: pore volume
API: American Petroleum Institute (gravity measurement)
Soi: initial oil saturation
Swi: initial water saturation
Sor: residual oil saturation
Kw: water permeability
Ko: oil permeability
mN/m: milliNewtons per meter (surface/interfacial tension unit)
mPa·s: milliPascal seconds (viscosity unit)
ppm: parts per million
mD: milliDarcy (permeability unit)
rpm: revolutions per minute
psi: pounds per square inch (pressure unit)
mol/kg: moles per kilogram
g/L: grams per liter
mL/min: milliliters per minute
**s** ^**–1**^: per second (shear rate unit)
**cm** ^**–1**^: per centimeter (wavenumber unit)
°C: degrees Celsius
cc: cubic centimeters
HMEL: HPCL-Mittal Energy Limited
DCAM: DCAM Engineering (equipment manufacturer)
